# 228 Physical literacy and inclusion within the context of disability and impairment: A multiple case study approach

**DOI:** 10.1093/eurpub/ckae114.098

**Published:** 2024-09-26

**Authors:** Kyle Pushkarenko, Jeffrey Crane, Jennifer Cowan

**Affiliations:** Memorial University Of Newfoundland, Canada; Memorial University Of Newfoundland, Canada; Memorial University Of Newfoundland, Canada

## Abstract

**Background and Purpose:**

Physical literacy (PL) holds significant importance in various organizational agendas, being prioritized as a core programming priority under the premise of inclusivity irrespective of ability. However, there is a notable gap in understanding PL within the context of disability and impairment, as application of PL often occurs through a lens of normativity and standardization by those who do not experience disability. This study aims to establish an evidence-based, context-specific and foundational understanding of PL and its inclusivity of all individuals regardless of ability level, that can be utilized to inform, guide, and support current and future programs.

**Methods:**

Employing the communities of practice theoretical model, a multiple case study design was utilized to delve into three purposefully selected cases rich in PL and inclusive practices. Data was collected through face-to-face, semi-structured interviews with stakeholders including participants, parents, practitioners, supervisors, and administrators. Supplemental data sources included direct observations, exploration of program documents, site photographs, and reflexive engagement. An inductive thematic analysis was conducted to uncover stakeholder perspectives, providing context-specific insights into PL as an inclusive concept.

**Findings:**

Three overarching themes emerged, elucidating the foundations of PL as an inclusive concept: environmental considerations, operating with intention, and ethical and informed practice. These themes intersect with one another to foster opportunities for PL development across age and ability spectrums.

**Conclusions:**

The findings offer evidence-based insights into PL and inclusion practices, offering a framework to move beyond normative and standardized notions of PL development.

**Support/Funding Source:**

Government of Canada Social Sciences and Humanities Research Council Partnership Engage Grant (File No. 892-2021-1009).

